# Distal tibial hypertrophic nonunion with deformity: treatment by fixator-assisted acute deformity correction and LCP fixation

**DOI:** 10.1007/s11751-012-0150-7

**Published:** 2012-10-27

**Authors:** Mahmoud A. El-Rosasy, Sameh A. El-Sallakh

**Affiliations:** Department of Orthopaedic Surgery and Traumatology, Faculty of Medicine, University of Tanta, Tanta, Egypt

**Keywords:** Tibia, Nonunion, Deformity, Internal fixation

## Abstract

Distal tibial hypertrophic nonunion with angular deformity has been successfully treated by circular external fixator. The inconvenience of the bulky external fixator and frequent pin tract infection would not be accepted in certain cases. This study included thirteen patients (mean age 39 years) with angular deformity of the distal dia-/metaphyseal tibial shaft. Five patients were originally treated by interlocking nail, three were treated by plate and screws fixation, four treated conservatively and one had deformity secondary to fracture of a lengthening regenerate. All patients were treated by osteotomy and acute correction of the deformity using temporary unilateral fixator and internal fixation by a locking compression plate (LCP). The external fixator was removed at the end of surgery. The results were evaluated both clinically and radiologically. All osteotomies healed within 3 (2–4) months. All patients were able to work within an average of 2.3 months. The function of the upper ankle joint was unrestricted in twelve cases, and in 1 case there was a mild functional deficit. The mean follow-up was 60 months (24–120). The frontal plane alignment parameters (the mechanical axis deviation, the lateral distal tibial angle and the medial proximal tibial angle) and the sagittal alignment parameters (the posterior proximal tibial angle and the anterior distal tibial angle) were within normal values postoperatively. No cases of deep infection or failure of fixation were encountered. Acute correction of distal tibial shaft hypertrophic nonunion with deformity and LCP fixation is a reliable option in well-selected cases.

## Introduction

Hypertrophic nonunions of the distal tibia that are associated with angular deformity have been successfully treated using circular external fixators [1]. The several advantages of gradual distraction of a hypertrophic nonunion reported include simultaneous correction of deformity and leg length discrepancy and achievement of bone consolidation [2]. However, the inconvenience of the bulky external fixator, joint stiffness and frequent pin tract infection are not acceptable to some patients [3, 4]. In contrast, while the use of intramedullary nails and conventional plates may not provide sufficiently rigid fixation for consolidation of the nonunion due either to the short distal bone segment or osteoporosis [5–10], the use of locking plate fixation may have overcome these limitations [11].

This study presents the results of treatment of selected hypertrophic nonunions of the distal tibia by acute correction of the deformity assisted by intra-operative use of unilateral external fixator and definitive fixation by a locking compression plate (LCP).

## Patients and methods

The material of this retrospective study included the cases of all the patients (13 patients) who were treated by the first author (MAE) in the period between February 2002 and December 2008. The charts and radiographs of the patients were reviewed by the second author (SAE) who was not involved in the surgical procedure. The patients’ ages ranged from 27 to 54 years (mean 39 years). There were eight males and five female patients. In five cases, the tibial fracture was originally treated by an interlocking nail, three were treated by plate and screws fixation, four treated conservatively and one was a nonunion and deformity secondary to fracture of a regenerate column from lengthening.

An associated angular deformity was present in all cases and ranged from 15° to 35° (mean 25°). The deformity was in the oblique plane except for six cases of standard (coronal or sagittal) plane deformity. Limb shortening was present in all cases and ranged from 1 to 2.4 cm (mean 1.6 cm). Previous infection was present in one case and resolved after debridement. There were no cases of active infection at the time of our treatment. The duration of nonunion ranged from eight to 23 months (mean 13 months). Table 1 presents the demographic data of the patients.

**Table 1:** Preoperative and postoperative data

Case	Age	Sex	Side	Previous treatment	Duration of nonunion (months)	Magnitude of deformity and apex direction		LLD (cm)	
						Preop	Postop	Preop	Postop
1	28	M	R	P & S	23	22° A-L	0°	2.4	2
2	54	F	R	IMN	19	15° L	0°	1	0
3	51	M	R	POP	21	22° A-L	0°	2.1	1.5
4	43	M	L	IMN	11	35° A	5° A	1.5	1
5	36	F	R	POP	8	27° L	0°	2.2	1.5
6	48	M	L	POP	16	28° A-L	0°	1	0
7	35	M	R	P & S	18	19° A-L	0°	1.5	0
8	46	M	R	IMN	22	23° A-L	0°	1	0
9	34	M	R	POP	17	15° A-L	0°	1.5	0
10	29	M	L	Reg. Fx.	8	30° M	0°	1	0
11	44	F	R	IMN	12	30° L	3° L	2	1
12	33	F	R	P & S	14	27° A-L	0°	1	0
13	27	F	R	IMN	20	30° M	4° L	1.5	0
Mean	39				13	25°	0.9°	1.6	0.5

M, male; F, female; R, right; L, left; P & S, plate and screws; IMN, Intramedullary nail; POP, plaster of Paris; Reg. Fx., regenerate fracture; A-L, anterolateral; L, lateral; A, anterior; M, medial; LLD, leg length discrepancy

The indications for the procedure were as follows:

1. Hypertrophic nonunion of the distal tibial shaft associated with deformity.
2. Absence of active infection.
3. The skin and soft-tissue conditions appropriate to allow surgical intervention and insertion of a medial plate.
4. The projected leg length discrepancy after deformity correction should be less than 2.4 cm.

The procedure was contraindicated if the patient had active infection, a large magnitude deformity (more than 35°), the need for leg lengthening and poor skin conditions.

### Preoperative evaluation

This included a complete history of the illness, especially of symptoms of current or previous infection and of smoking. The involved limb was examined for evidence of a prior or actively draining sinus and erythema or indurations of the skin. Preoperative laboratory tests included a complete blood count (CBC), C-reactive protein (CRP) and erythrocyte sedimentation rate (ESR).

**Fig. 1:**
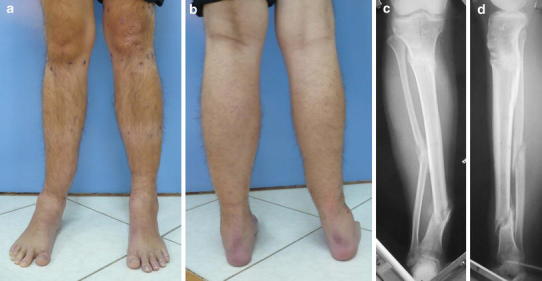
**a**, **b** preoperative clinical photos show angular deformity of the lower leg due to fracture of a lengthening regenerate; **c**, **d** preoperative x-rays show hypertrophic nonunion of the lower third of the tibia with medial angulation

The radiographic examination included radiographs of the affected and healthy sides, in the true antero-posterior and lateral planes for deformity analysis and preoperative planning [12, 13] (Fig. 1). No scanogram or full-length radiographs were done.

### The procedure

**Fig. 2:**
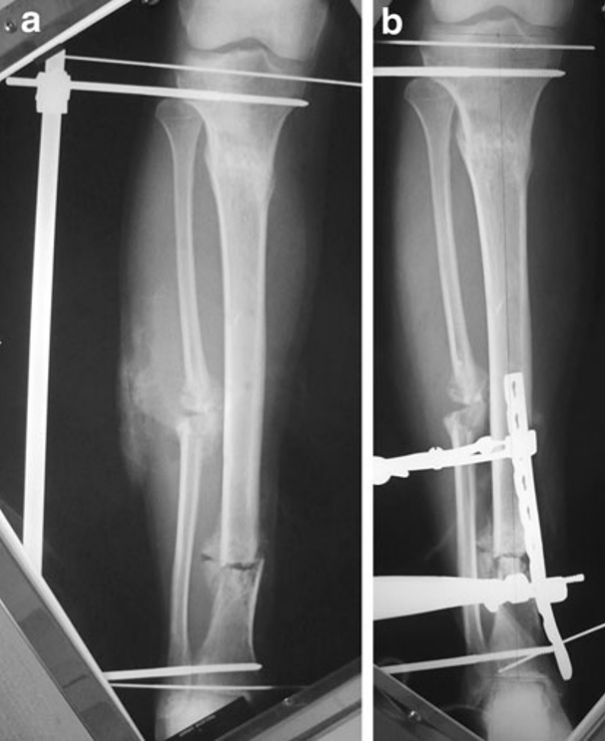
**a**, **b** intra-operative radiographs after deformity correction and preliminary plate positioning

A pneumatic tourniquet was applied to the upper thigh. An osteotomy of the fibula was performed at the apex of the deformity through a lateral approach to the fibula, multiple drill holes and completed manually with an osteotome. In cases of correction of a varus deformity, the fibular osteotomy ends were displaced to allow correction of tibial varus deformity. Under image intensifier control, the limb is rotated so that the plane of maximum tibial deformity profile was identified. With the limb held in this fashion, a 6-mm half pin was inserted in the tibia proximal to the proposed osteotomy and orthogonal to the anatomic axis of the proximal segment, with a second similar pin inserted distal to the proposed osteotomy parallel to the joint line of the ankle and orthogonal to the anatomic axis of the distal segment. The half pins and the external fixator bar were applied on the lateral aspect of the leg to avoid being an obstacle for medial plate application. Multiple drill holes were performed at the apex of the tibial deformity perpendicular to the axis of deformity correction. Over-drilling of the cortex on the convex side of the deformity was done using a larger drill bit to create a partial closing wedge osteotomy and facilitate acute correction of the deformity without acute stretching of soft tissues on the concave side. The deformity was then corrected using the half pins as cantilevers and the fixator bar connected to the pins to maintain the corrected position. At that point, a hard copy X-ray film was obtained for the measurement of mechanical axes, medial proximal tibial angle (MPTA) and lateral distal tibial angle (LDTA) (Fig. 2). A sharp bone chisel was used to remove excess bone off the medial surface of the tibia and to create a flat and even surface to accept the plate. These bone chips were saved to close any resultant defect (local autograft). A LCP of suitable length was contoured to match the medial surface of the distal tibia and temporarily held in place by Kirschner wires. Under image intensifier, the level of the distal-most screws of the plate was confirmed by a Kirschner wire to avoid intra-articular penetration of the screws (Fig. 2). One cortical 4.5-mm screw was inserted on either side of the osteotomy in the dynamic compression unit (DCU) of the combination hole to create axial compression of the osteotomy site and close bone gaps. The remaining screws were inserted in the threaded locking holes using locking screws. The wound was copiously irrigated, and the wound was closed in layers. No external support or cast was used.

### Postoperative management

**Fig. 3:**
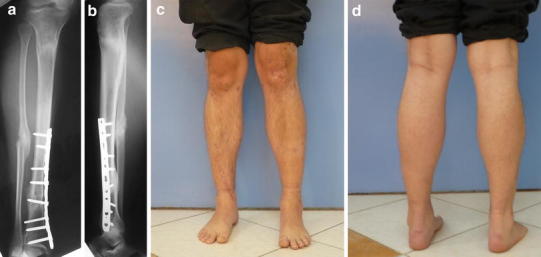
**a**–**d** follow-up radiographs and clinical photographs after consolidation of the nonunion with restoration of limb alignment

Touchdown weight bearing was allowed a few days postoperatively when pain and edema had subsided. Weight bearing was then gradually increased as bone consolidation progressed (Fig. 3).

### Evaluation of results

The results of treatment were evaluated using the evaluation system described by Paley et al. with modification [14]. The evaluation included the following clinical (objective and subjective) and radiological parameters: bone union, residual deformity, residual leg length discrepancy (LLD), recurrent infection, soft-tissue healing, joint contracture, persistent pain, whether the patient was able to return to their previous occupation, and the patient’s satisfaction with the result of the procedure. For a satisfactory result, the aforementioned criteria should be fulfilled. Failure to achieve any of these criteria indicated an unsatisfactory result.

## Results

The follow-up ranged from 24 to 120 months (mean 60 months) after bone healing. All osteotomies healed within 2–4 months (mean 3 months). All patients were able to return to work within a mean of 2.3 months. The function of the ankle joint was unrestricted in twelve cases, but in one case, there was a mild functional deficit due to preoperative ankle stiffness which did not resolve with physiotherapy. The frontal plane alignment parameters (the mechanical axis deviation, the lateral distal tibial angle and the medial proximal tibial angle) and the sagittal alignment parameters (the posterior proximal tibial angle and the anterior distal tibial angle) were within normal values postoperatively [12, 13]. No cases of deep infection or failure of fixation were encountered. Bone grafting was not needed in any case. A residual leg length discrepancy of less than 2.4 cm was present in five cases. All patients were satisfied with the functional outcome except for one patient who requested correction of a 2-cm leg length discrepancy; tibial lengthening over the plate was carried out later.

### Complications

Surgery to remove the plate due to skin irritation due to the subcutaneous location of the plate was needed in two cases. In two cases, a peroneal nerve palsy (paresthesia on the dorsum of the foot) developed after correction of 35° of medial angulation. The nerve palsy improved in 3 weeks with no residual deficit. No other cases of nerve or vascular injury were encountered as a result of this treatment.

## Discussion

The development of a mal-aligned nonunion after a tibial fracture produces alterations in joint reaction forces and increases shear stresses on the articular cartilage of adjacent joints; this may lead to degenerative arthritis. In addition, the presence of deformity leads to alterations in gait, lower extremity mechanics, functional impairment and has a poor cosmetic appearance [15].

The goals in the management of a nonunion of the tibia with deformity include achievement of union with restoration of the length, alignment and function of the limb. Operative approaches for the treatment of hypertrophic nonunions of the tibial diaphysis with autogenous bone graft alone or in conjunction with fixation with a conventional plate or intramedullary nails have been supplemented by the documented success of distraction histogenesis of the nonunion using circular external fixators [1, 2]. Despite this success, some patients would not be candidates for this line of treatment with difficulties in the use of the external fixation device, particularly from poor patient tolerance and pin site infections.

McKee and colleagues demonstrated a marked improvement with respect to the patient’s general health status after treatment using the Ilizarov device to correct post-traumatic lower-limb deformity. In the same study, they noted that the patients’ health status remained well below normal population means even at 2 years after completion of treatment. Complications in the course of treatment of nonunions with the Ilizarov device were common. In particular, the effect of the Ilizarov device on adjacent joints during distraction is of concern as it was shown to increase the pressure on articular cartilage. Joint stiffness is another well-recognized complication [3].

In the study by Sanders et al. [4], they demonstrated that although the nonunion can be successfully healed using the techniques of Ilizarov, residual morbidity remains significant at a mean follow-up of 39 months. Ninety percent of their patients had evidence of ankle dysfunction or arthritis. They gave several possible explanations for this finding of which one was the use of a ring fixator around the distal tibia results in ankle stiffness, with subsequent development of dysfunction and joint space narrowing.

The use of locking compression plate (LCP) combines the convenience of internal fixation and stability of an internal fixator [16]. In contrast to external fixation techniques, the long learning curve is short and shares much with the basic techniques of internal fixation. A shortcoming of this treatment is that it does not allow correction of leg length discrepancy although it can be addressed using the technique of leg lengthening over the plate; this loses the advantage of avoiding use of an external fixator in the postoperative period. Another limitation of this study is the small number of cases in the series; this is attributed to the indications for patient selection.

## Conclusion

This case series suggests that external fixator-assisted acute corrections of deformities associated with distal tibial hypertrophic nonunions can be performed safely and successfully. The indications of this series suggest deformities no greater than 35° and with an anticipated post-correction length discrepancy of less than 2.4 cm. The use of a medial locking compression plate was associated with few complications and proved a suitable device to achieve bone union.
